# Supplementary material for: Synergistic Phototherapy-Molecular Targeted Therapy Combined with Tumor Exosome Nanoparticles for Oral Squamous Cell Carcinoma Treatment
Source: Pharmaceutics. 2023 Dec 26;16(1):33. doi: 10.3390/pharmaceutics16010033 (PMC10821490; doi:10.3390/pharmaceutics16010033)
Supplement: Supplementary file 1 [file pharmaceutics-16-00033-s001.zip › pharmaceutics-2711323-supplementary.pdf]

# Synergistic Phototherapy-Molecular Targeted Therapy Combined with Tumor Exosome Nanoparticles for Oral Squamous Cell Carcinoma Treatment

**Ming Li[1†], Shiyao Yin[2†], Anan Xu[1†], Liyuan Kang[1], Ziqian Ma[1], Fan Liu[1], Tao Yang[1], Peng Sun[2,*] and Yongan Tang[1,*]**

[1] Jiangsu Key Laboratory of Neuropsychiatric Diseases, and College of Pharmaceutical Sciences, Soochow University, Suzhou 215123, China.; mli0919@suda.edu.cn (M.L.); 20214226024@stu.suda.edu.cn (A.X.); 20224226022@stu.suda.edu.cn (L.K.); 20235226014@stu.suda.edu.cn (Z.M.); liufan@suda.edu.cn (F.L.); tyang0920@suda.edu.cn (T.Y.)

[2] Department of Otolaryngology, the First Affiliated Hospital of Soochow University, Suzhou 215006, China.; 20215232268@stu.suda.edu.cn (S.Y.)

* Correspondence: sunpeng@suda.edu.cn (P.S.); yatang@suda.edu.cn (Y.T.)

† These authors contributed equally to this work.

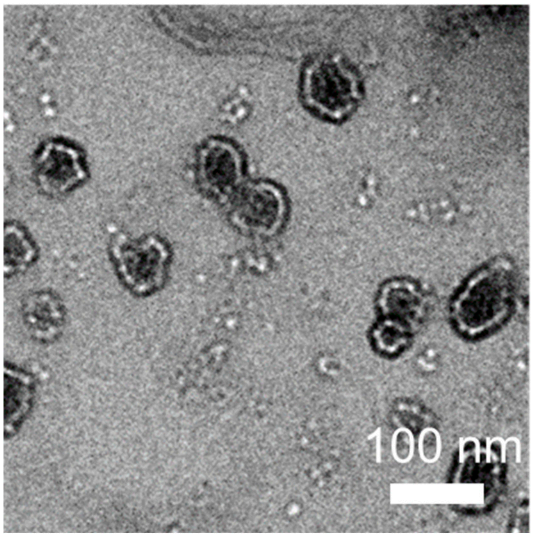


Figure S1. TEM image of blank EXOs.

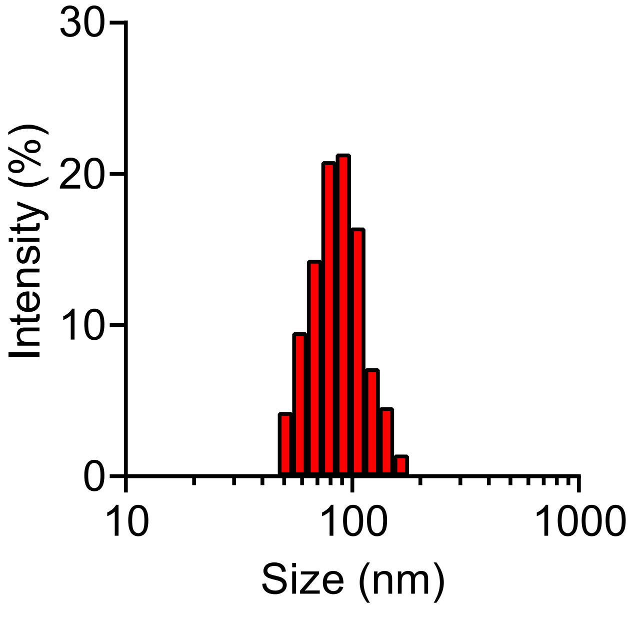


Figure S2. DLS measurement of blank EXOs.

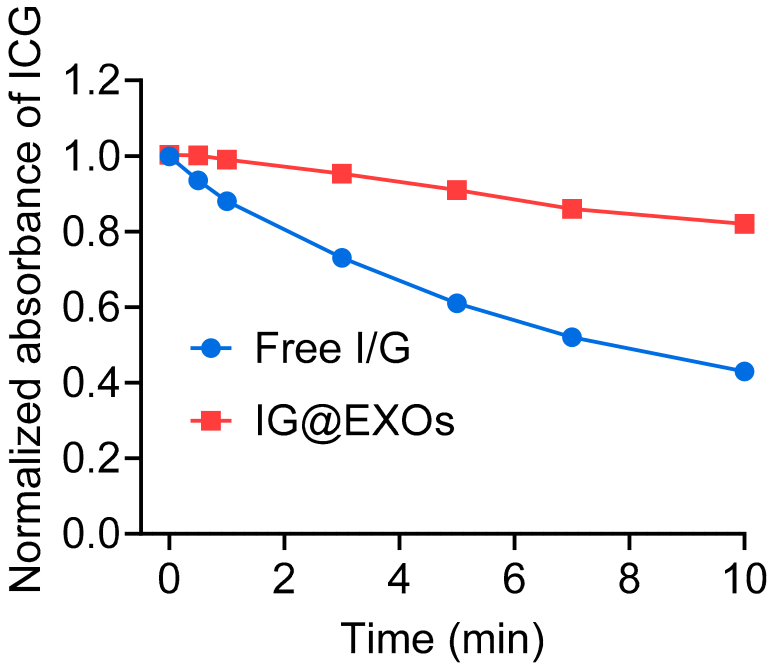


Figure S3. Normalized absorbance of ICG at 785 nm in the solutions of Free I/G or IG@EXOs under laser irradiation (785 nm, 0.5 W $cm^{-2}$) at different time points.

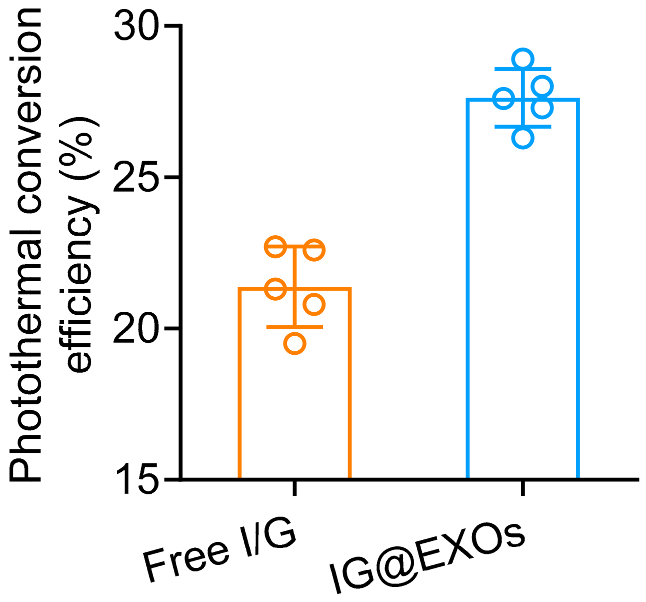


Figure S4. Photothermal conversion efficiency of Free I/G and IG@EXOs with laser irradiation (785 nm, 0.5 W $cm^{-2}$).

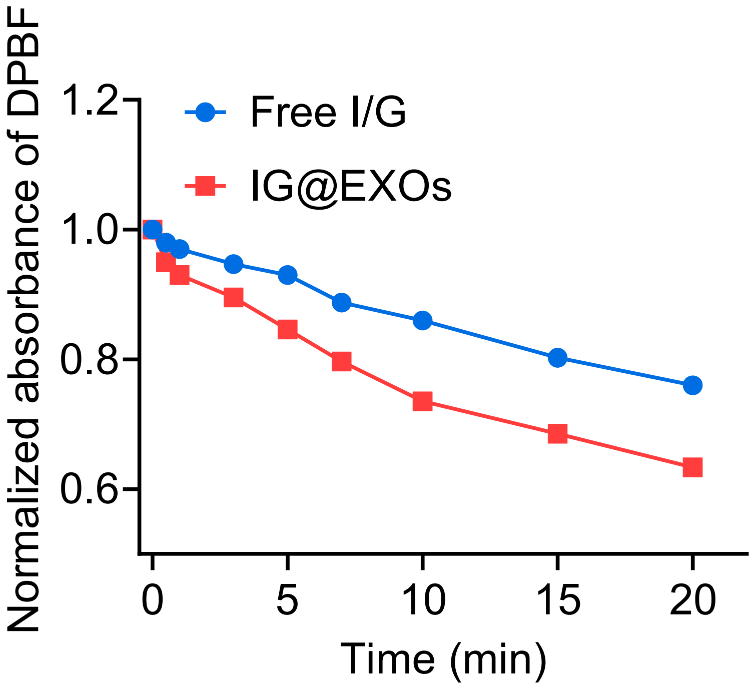


Figure S5. Normalized absorbance of DPBF at 415 nm in the solutions of Free I/G or IG@EXOs under laser irradiation (785 nm, 0.5 W $cm^{-2}$) at different time points.

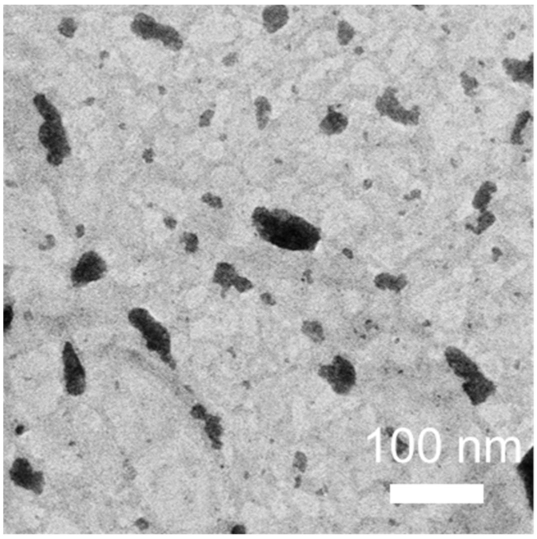


Figure S6. TEM image of IG@EXOs after 785 nm laser irradiation (0.5 W $cm^{-2}$, 5 min).

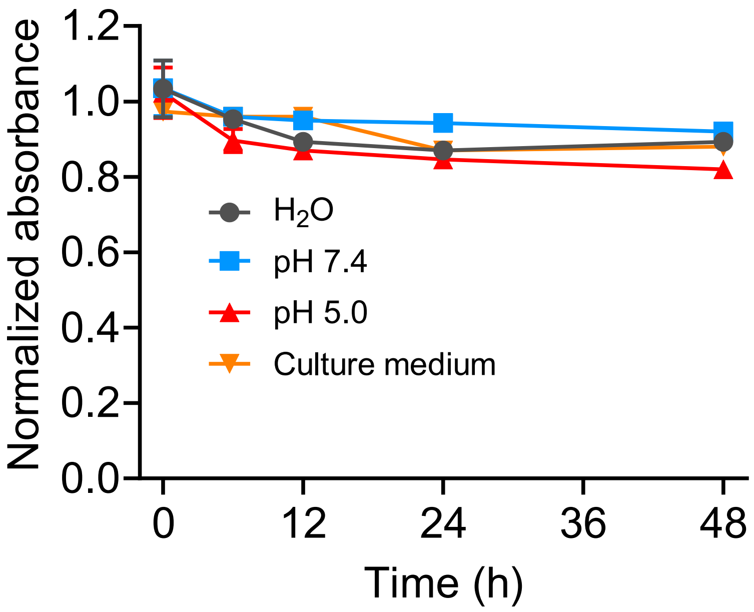


Figure S7. Normalized absorbance of IG@EXOs at 785 nm in various solvents.

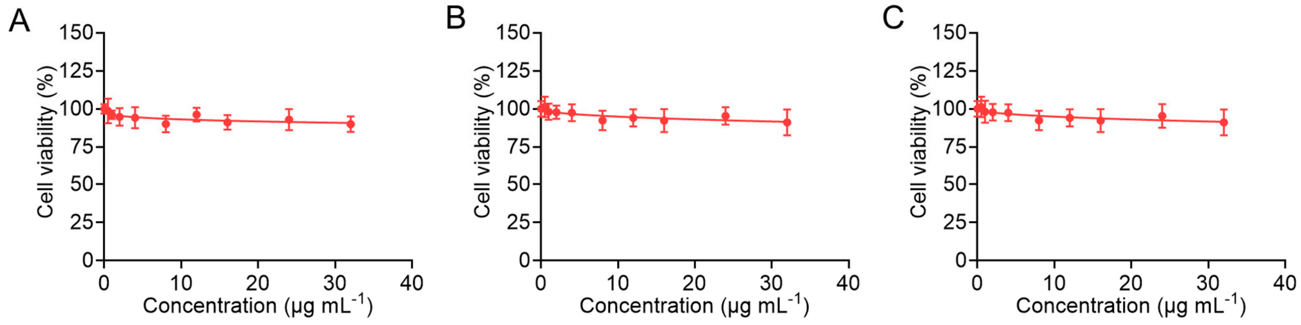


Figure S8. Cell viability of (A) L929, (B) 293T, and (C) HUEVC cells treated with IG@EXOs at various concentrations.

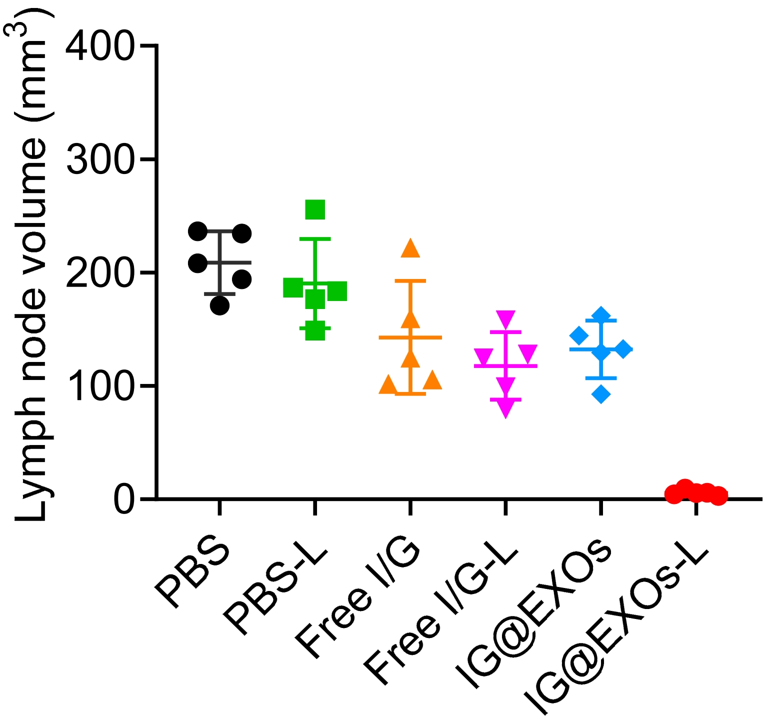


Figure S9. Volume of the lymph nodes from mice treated with different formulations.

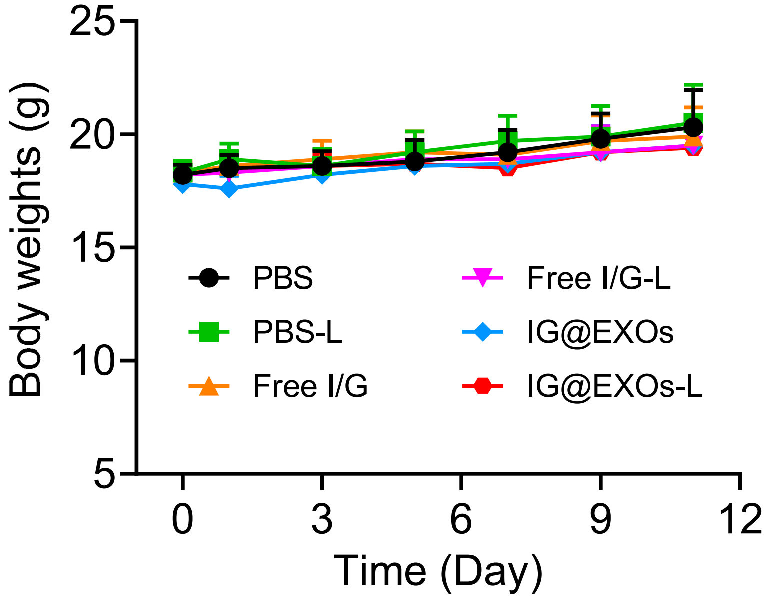


Figure S10. Body weights of mice treated with different formulations.
